# Elucidation of the mechanism of human growth hormone-Fc fusion protein in accelerating tibial fracture healing in rats via untargeted metabolomics

**DOI:** 10.3389/fmolb.2026.1971992

**Published:** 2026-10-28

**Authors:** Jianyang Gu, Juntong Liu, Binghuai Peng, Baisong Zhou, Zhuonan Li, Hao Zhang, Tianyu Zhang, Haoming Jiang, Ruixin Sun, Ping Gong, Han Liu

**Affiliations:** 1 Changchun University of Science and Technology, Changchun, China; 2 Changchun Institute of Biological Products Co., Ltd., Changchun, China

**Keywords:** fracture healing, growth hormone, mechanism, metabolomics, rat tibial fracture model

## Abstract

**Background:**

Fracture healing has always been a major challenge in clinical practice. This study aims to explore the effect of human growth hormone-Fc fusion protein on the healing of a rat tibial fracture model and its possible mechanisms of action.

**Methods:**

By assessing the levels of growth hormone effectors IGF-1 and IGFBP-3 in rats, combined with Micro-CT scanning and pathological staining slices, we investigated the pathological effects of subcutaneous injection of human growth hormone-Fc fusion protein for 28 days on the healing of rat tibial fractures. Serum metabolomics studies were conducted using the AB Triple TOF 6600 mass spectrometer to further investigate the bio-metabolites relevant to fracture healing influenced by the human growth hormone-Fc fusion protein.

**Results:**

The study showed that after administration of the growth hormone-Fc fusion protein, IGF-1 and IGFBP-3 levels in rat blood were significantly increased. Micro-CT scans and pathological staining also demonstrated that the high-dose group had a marked promoting effect on fracture healing, with a healing rate significantly superior to the positive drug group; the medium and low dose groups also showed certain degrees of promotion. Metabolomic results revealed that the growth hormone could regulate 17 metabolites, affecting 8 metabolic pathways in relation to fracture healing.

**Conclusion:**

The study found that the growth hormone-Fc fusion protein shortened the healing period of the rat tibial fracture model by more than 1 week, possibly by influencing the levels of IGF-1 and IGFBP-3 factors, as well as regulating 17 metabolites in 8 metabolic pathways—including starch and sucrose metabolism, galactose metabolism, glycerophospholipid metabolism, terpene backbone biosynthesis, glyoxylate and dicarboxylate metabolism, retinol metabolism, glycine, serine, and threonine metabolism, and inositol phosphate metabolism—thus promoting fracture healing. This provides a basis for subsequent larger-scale, multicenter experiments and clinical translational research on FcGH in the treatment of fractures.

## 1 Introduction

Fractures are a common traumatic injury of the musculoskeletal system, causing pain and limited mobility for patients, bringing economic burdens, and seriously affecting quality of life (Cooper, 1997). Studies have shown that the risk of fractures is higher among the elderly population (Foulke et al., 2016). Factors such as decreased immune function, inflammation, stem cell exhaustion and dysfunction, and reduced angiogenic capacity lead to slower healing after fractures in the elderly (Clark et al., 2017). Fracture healing is a highly coordinated physiological process and also a repetition of bone development, which, as shown in Figure 1, can be divided into several consecutive stages. The first is the inflammatory phase: blood vessels rupture, forming a hematoma, in which immune cells release various factors to accelerate the aggregation of mesenchymal stem cells (MSCs), form granulation tissue, and induce neovascularization (Hixon and Miller, 2022). Next is the reparative phase: mesenchymal stem cells begin to differentiate, gradually forming cartilage tissue, providing initial stabilization at the fracture site. Meanwhile, various cellular factors continue to promote angiogenesis, and under their combined effects, the subperiosteal cartilage continuously thickens (Knox et al., 2021). A cells further differentiate into osteoblasts and osteoclasts and undergo apoptosis, cartilage gradually forms a cartilage callus and begins to undergo endochondral ossification. Under the periosteum, woven bone continues to be deposited and calcified, eventually forming a hard bony callus (Sheen et al., 2026). In the remodeling phase, osteoclasts gradually resorb the bone matrix and cooperate with osteoblasts, leading to continuous remodeling of the bony callus—a process known as the coupling cycle. After months or even years of reconstruction, the center of the callus gradually transforms into compact bone, while the edges gradually form lamellar bone, ultimately restoring the structure and function of the bone. This process is also known as coupled remodeling (Einhorn and Gerstenfeld, 2014; Sheen et al., 2026).

**FIGURE 1:**
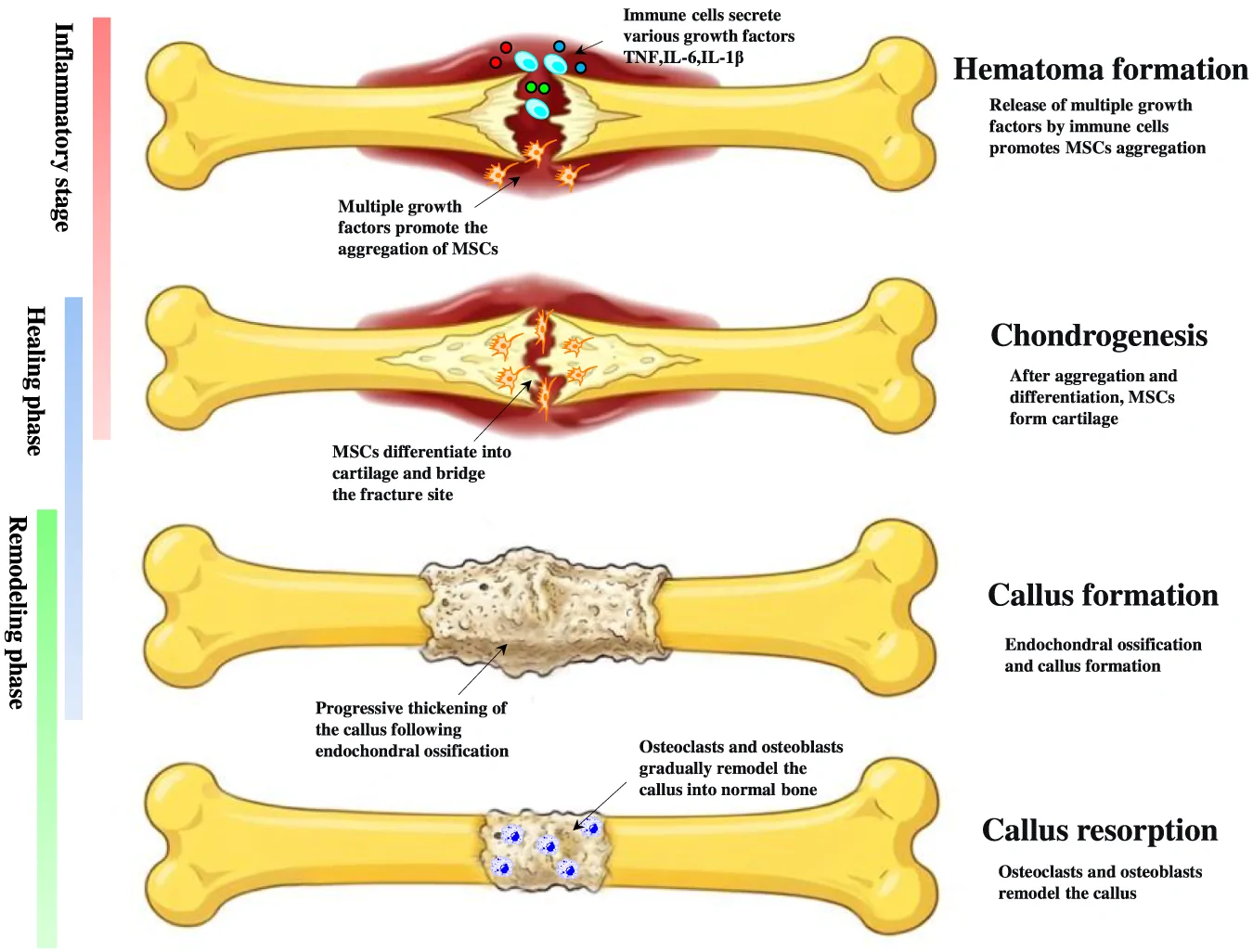
Schematic diagram of fracture healing.

Studies have shown that multiple drugs, including pentoxifylline, nebivolol, and vardenafil, can accelerate fracture healing, as evidenced by a series of animal experiments, through the improvement of the nitric oxide pathway and increased blood flow (Atalay et al., 2015; Metineren et al., 2017; Atcı et al., 2021); The channel blocker amlodipine can promote bone formation and angiogenesis while accelerating callus remodeling (Menger et al., 2021);curcumin increases the number of osteoblasts by activating the autophagy pathway (Li et al., 2018); the anti-osteoporosis drug romosozumab has shown good effects in promoting fracture healing, but none have shown clinical results (Chandran et al., 2024).

Currently, drugs clinically used to treat fractures mainly include anti-resorptive drugs and bone-promoting drugs. Anti-resorptive drugs, represented by bisphosphonates, enhance bone density by inhibiting the bone resorption activity of osteoclasts, thus reducing the risk of fractures in patients, but they can also cause side effects such as atypical femoral fractures (Bellido, 2024; Agarwal et al., 2026). Bone-promoting drugs represented by teriparatide can significantly promote endochondral ossification, increase bone density, and accelerate fracture healing, without increasing adverse events (Luo et al., 2024).They are also used in clinical practice to promote postoperative fracture healing (Barvencik, 2015; Selvakumar et al., 2026). In this experiment, teriparatide was used as the positive control drug.

Human growth hormone (GH) was initially mainly used to treat children with growth hormone deficiency (GHD) (Maghnie et al., 2022).In the following decades of research, it was found to be effective for various diseases. Currently, the indications for pediatric growth hormone approved by the U.S. Food and Drug Administration mainly include eight types: growth hormone deficiency (GHD), Prader-Willi syndrome (PWS), small for gestational age (SGA) without catch-up growth, idiopathic short stature (ISS), Turner syndrome (TS), SHOX gene haploinsufficiency, Noonan syndrome, and chronic renal insufficiency (Danowitz and Grimberg, 2022).

As a hormone drug, GH has a relatively short half-life in the human body, so reducing the metabolic rate of GH in the body has become a main research focus. PEG-rhGH is a relatively mature method; binding with PEG can effectively prolong the half-life of GH, but some side effects exist (Wang et al., 2025). HAS-GH fusion protein also prolongs the half-life of GH, but reduces its bioactivity (Ding et al., 2014). Our previous research showed that recombinant FcGH fusion protein, which fuses GH with the naturally occurring human Fc protein, can effectively reduce the risk of immune reactions and significantly prolong the duration of action of GH. FcGH powerfully stimulates effector molecules insulin-like growth factor-1 (IGF-1) and IGF binding protein-3 (IGFBP-3) (Liu et al., 2025). IGF-1, produced mainly by the liver and widely distributed in the human brain and skeletal muscle, plays an important role in promoting bone growth (Bikle et al., 2015).Insulin-like Growth Factor Binding Protein (IGFBPs) are the main binding targets of IGF-1 and include six subtypes, IGFBP-1 through IGFBP-6. Among them, IGFBP-3 can bind to 75% of IGF-1 in plasma and is the major binding protein of IGF-1 (Locatelli and Bianchi, 2014). Studies have shown that IGF-1 and IGFBP-3 factors affect the process of bone development (Xian et al., 2012; Bikle et al., 2015).

In this study, the promotive effect of GH on fracture healing was evaluated using a rat tibial fracture model, and its mechanism was explored based on serum metabolomics research.

## 2 Results

### 2.1 Effects on rats with tibial fracture

#### 2.1.1 Results of IGF-1 and IGFBP-3 content detection

As shown in Figure 2, at 4 weeks post-dosing, the IGF-1 and IGFBP-3 levels in the Model group differed significantly from those in the Control group, and the levels in the FcGH high-, medium-, and low-dose groups were also significantly different from those in the Model group.

**FIGURE 2:**
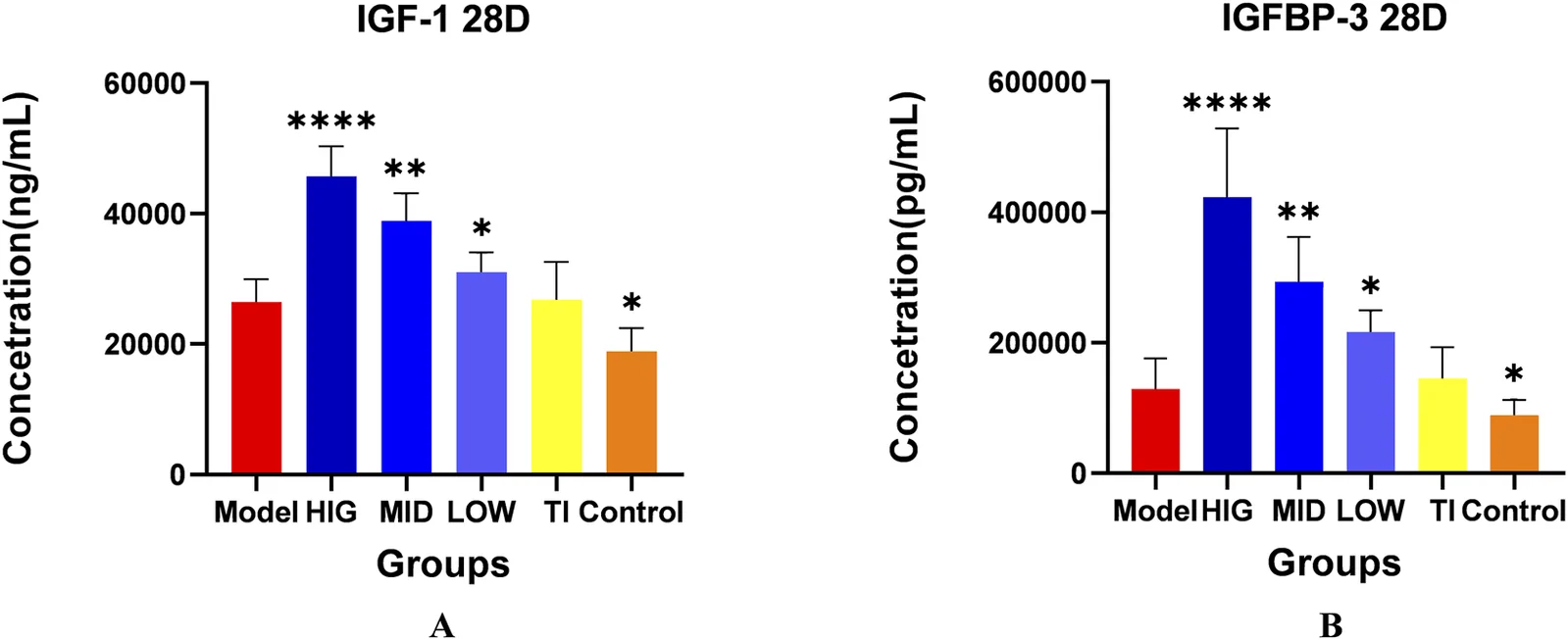
**(A)** IGF-1, **(B)** IGFBP-3, mean ± standard deviation (n = 4), compared with the Model group****p < 0.0001, **p < 0.01, *p < 0.05.

#### 2.1.2 Micro-CT scan detection results

As shown in Figure 3, CT results indicate that after 2 weeks of administration, all treatment groups exhibited obvious callus formation, suggesting that all groups promoted fracture healing. After 3 weeks of administration, the high-dose FcGH group no longer showed a visible fracture line, whereas the other groups still had clearly visible fracture lines. After 4 weeks of administration, the TI group and both the medium- and high-dose FcGH groups showed no obvious fracture line, and the callus had significantly thickened; in the high-dose group, the callus had decreased in size and was nearly completely healed. The results demonstrate that the fracture-healing effect of FcGH is dose-dependent, with the high-dose group exhibiting a healing period shortened by more than 1 week compared to the TI group, and by about 1 week or more shorter than the fracture-healing period reported in preliminary experiments and the literature for rats (Desai et al., 2003).

**FIGURE 3:**
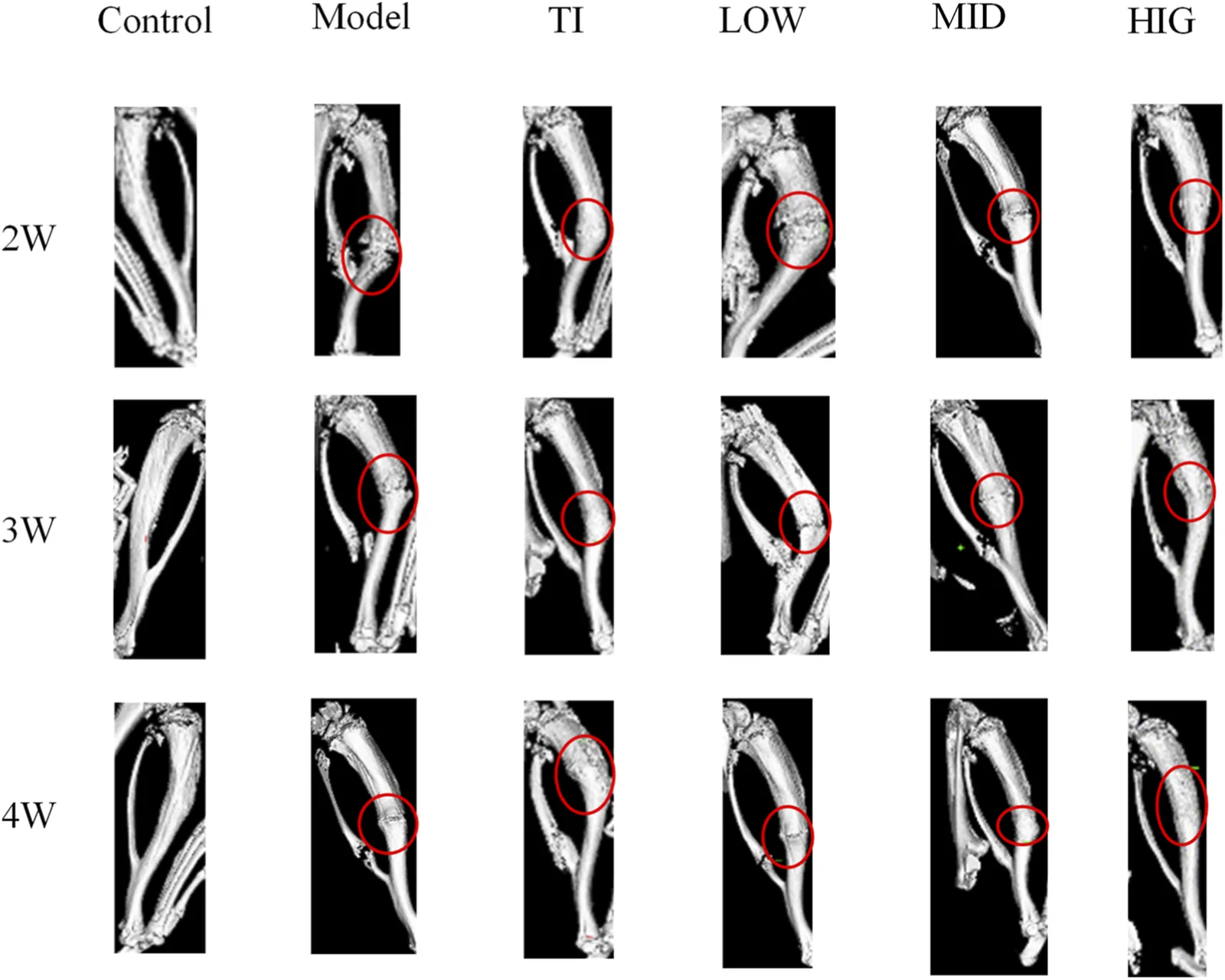
CT scan images of rat tibias at 2 weeks, 3 weeks, and 4 weeks.

#### 2.1.3 Safranin O-Fast green histopathological staining

As shown in Figure 4, the safranin O–fast green histological staining indicated that after 2 weeks of administration, all groups displayed obvious fracture gaps. In the drug-treated groups, the red cartilage tissue surrounding the fracture gaps was more pronounced compared to the model group, suggesting that all drug-treated groups could promote fracture healing. After 3 weeks of administration, there were no obvious fracture gaps in both the high-dose FcGH group and the TI group; however, cartilage tissue had disappeared in the high-dose group, whereas the TI group still had some cartilage tissue. Obvious fracture gaps could still be observed in the remaining drug-treated groups, indicating that the high-dose FcGH group showed the best efficacy in promoting fracture healing. After 4 weeks of administration, cartilage tissue had disappeared in both the high-dose FcGH group and the TI group, and osteocytes were dense and well-organized. The medium- and low-dose groups still retained a small amount of cartilage tissue, with the area of cartilage in the low-dose group being notably larger, suggesting that FcGH’s effect on promoting fracture healing is dose-dependent. The results showed that FcGH is more effective than teriparatide in promoting fracture healing and exhibits a dose-dependent effect, with pathological findings consistent with imaging results.

**FIGURE 4:**
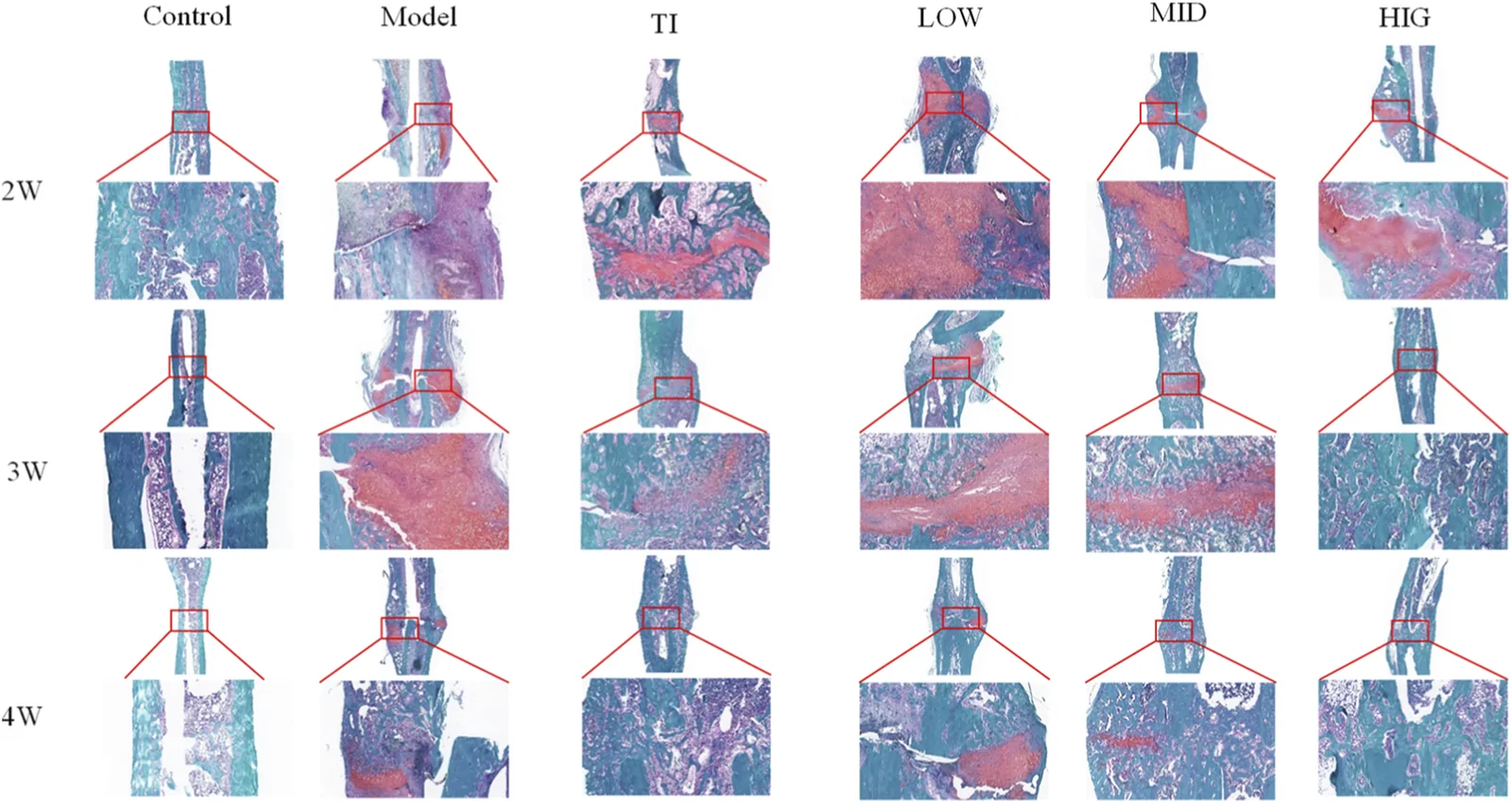
Histopathological staining with Safranin O-Fast Green on rat tibial sections at 2, 3, and 4 weeks.

### 2.2 Metabolomics research

#### 2.2.1 Experimental data quality evaluation

The total ion chromatograms (Total Ion Chromatogram, TIC) of the QC samples were overlapped and compared, as shown in Figures 5A,B. The response intensities and retention times of the chromatographic peaks basically overlap, indicating that the variation caused by instrument error throughout the experiment was minimal. Pearson correlation analysis was performed on the QC samples, as shown in Figure 5C. Generally, a correlation coefficient greater than 0.9 indicates good correlation. The results show that the correlation coefficients between QC samples are all above 0.9, indicating good experimental repeatability. The smaller the relative standard deviation (RSD) of ion peak abundance in QC samples, the better the stability of the instrument, which is an important indicator reflecting data quality. The proportion of peaks with RSD≤30% in QC samples accounts for more than 70% of the total number of peaks in QC samples, as shown in Figure 5D, indicating that the instrument analysis system is relatively stable and the data can be used for subsequent analysis.

**FIGURE 5:**
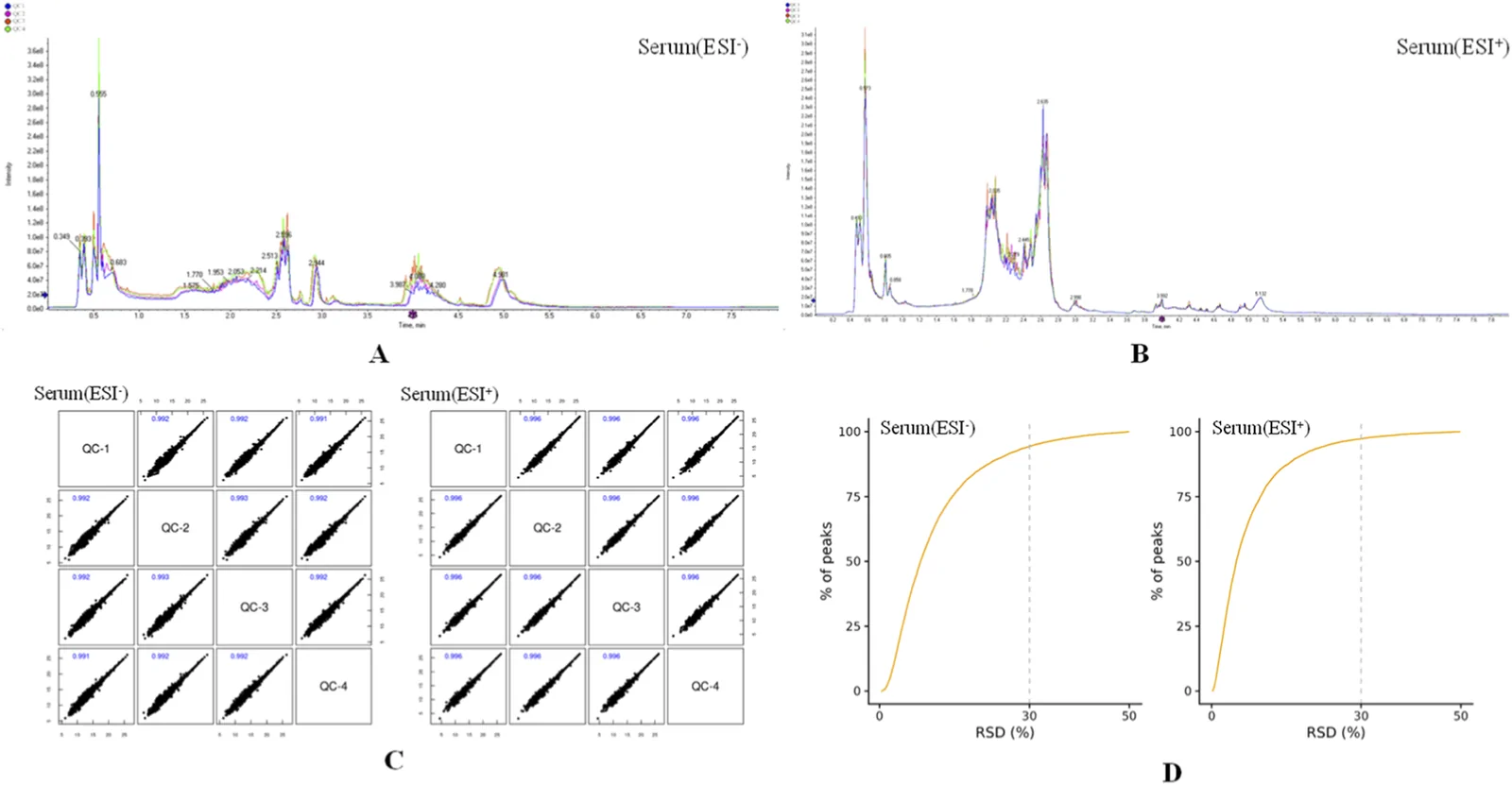
**(A)** Total ion chromatogram (TIC) of QC samples in ESI- mode, **(B)** Total ion chromatogram (TIC) of QC samples in ESI + mode, **(C)** QC sample correlation map, **(D)** QC sample relative standard deviation (RSD).

#### 2.2.2 Multivariate statistical analysis of serum metabolomics

Principal component analysis (PCA) was performed in both ESI+ and ESI− modes. The metabolite profile PCA of serum samples is shown in Figure 6A, where different groups are represented by dots of different colors. All QC samples are clustered in the central region, demonstrating the system’s stability and reliability. The samples from the Control, Model, and FcGH groups are located in different regions and exhibit distinct clustering characteristics, indicating metabolic differences among the three groups.

**FIGURE 6:**
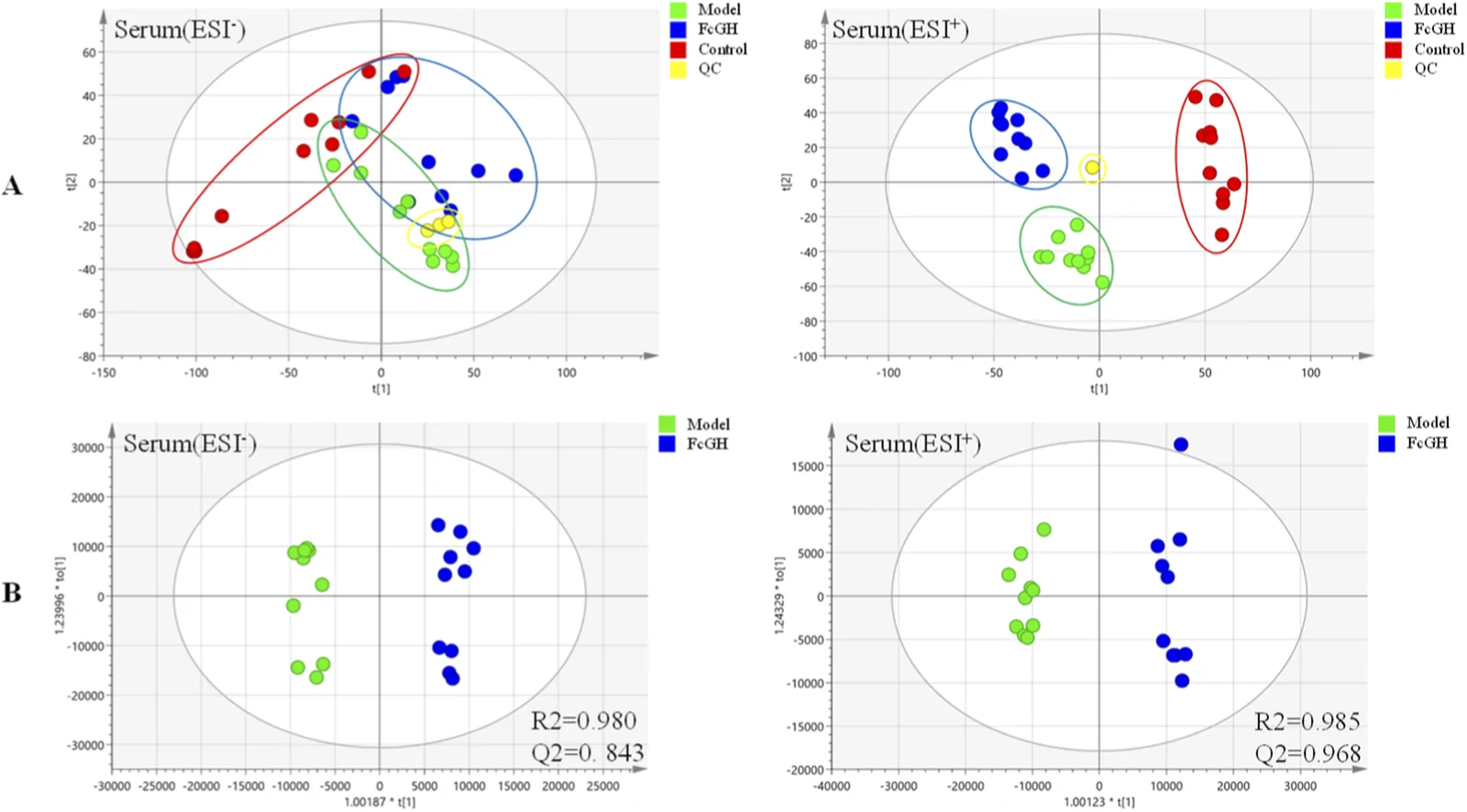
**(A)** Principal component analysis (PCA), **(B)** OPLS-DA model.

Using orthogonal partial least squares discriminant analysis, an OPLS-DA model was established. As shown in Figure 6B, there is a clear separation and clustering between the Model group and FcGH group samples. The explanatory parameter R2 and the predictive parameter Q2 are both close to 1, indicating that the model is reliable and has good predictive ability. The model was further validated by 200 permutation tests, and the results are shown in Figure 7A. The original points on the right are consistently higher than all randomly generated R2 and Q2 values on the left, indicating that the model’s predictive ability is quite accurate. The volcano plot provides a clearer representation of the differential metabolites, as shown in Figure 7B, where green dots indicate downregulated metabolites (FC < 0.5), red dots indicate upregulated metabolites (FC > 2), and blue dots represent the 17 identified differential metabolites.

**FIGURE 7:**
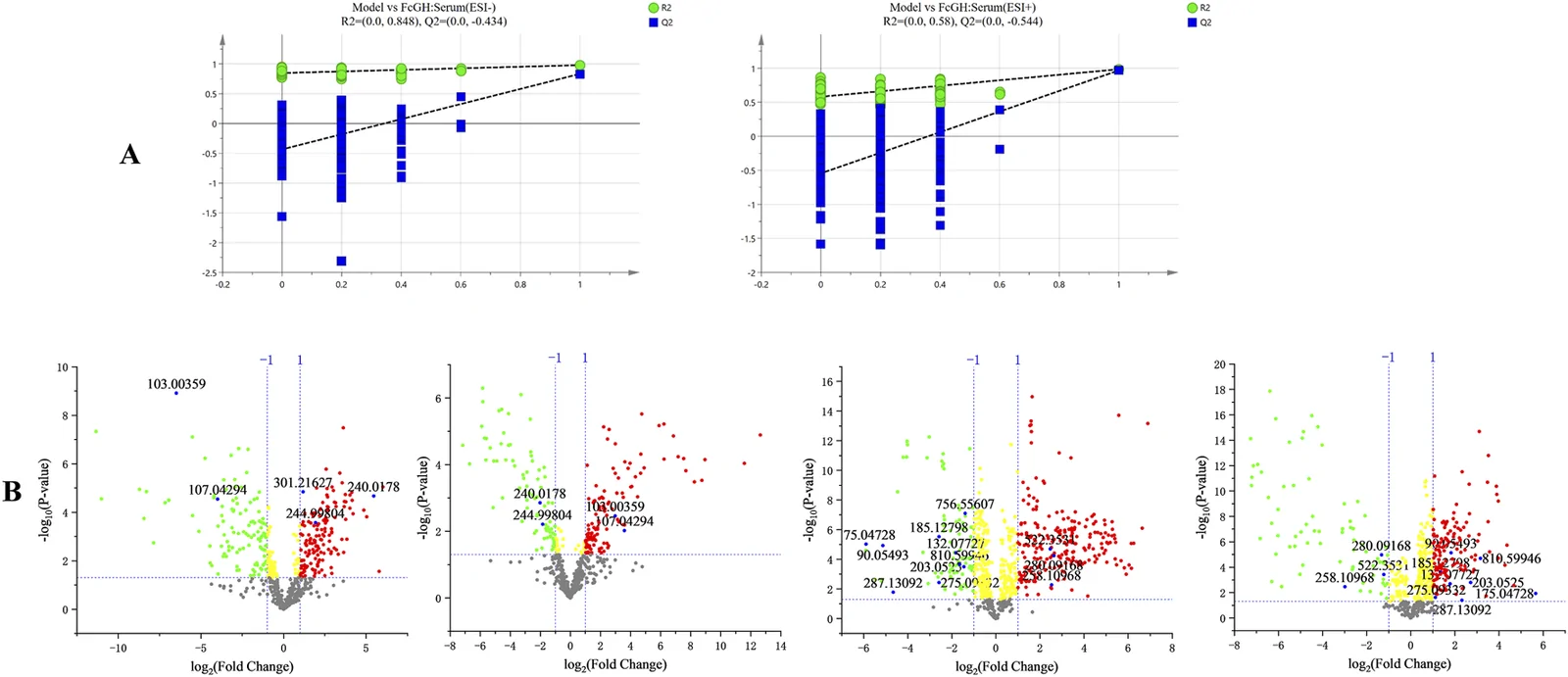
**(A)** Permutation test, **(B)** Volcano plot.

Receiver operating characteristic curves (ROC) were generated for 17 differential metabolites, as shown in Figure 8. The area under the curve (AUC) and p-values for these 17 differential metabolites are presented in Table 1. All differential metabolites had an AUC greater than 0.8 and p-values less than 0.05. The results from the Model group and Control group indicate that all 17 metabolites are associated with fracture healing. The results from the Model group and FcGH group show that FcGH indeed affects fracture healing by influencing these 17 metabolites.

**FIGURE 8:**
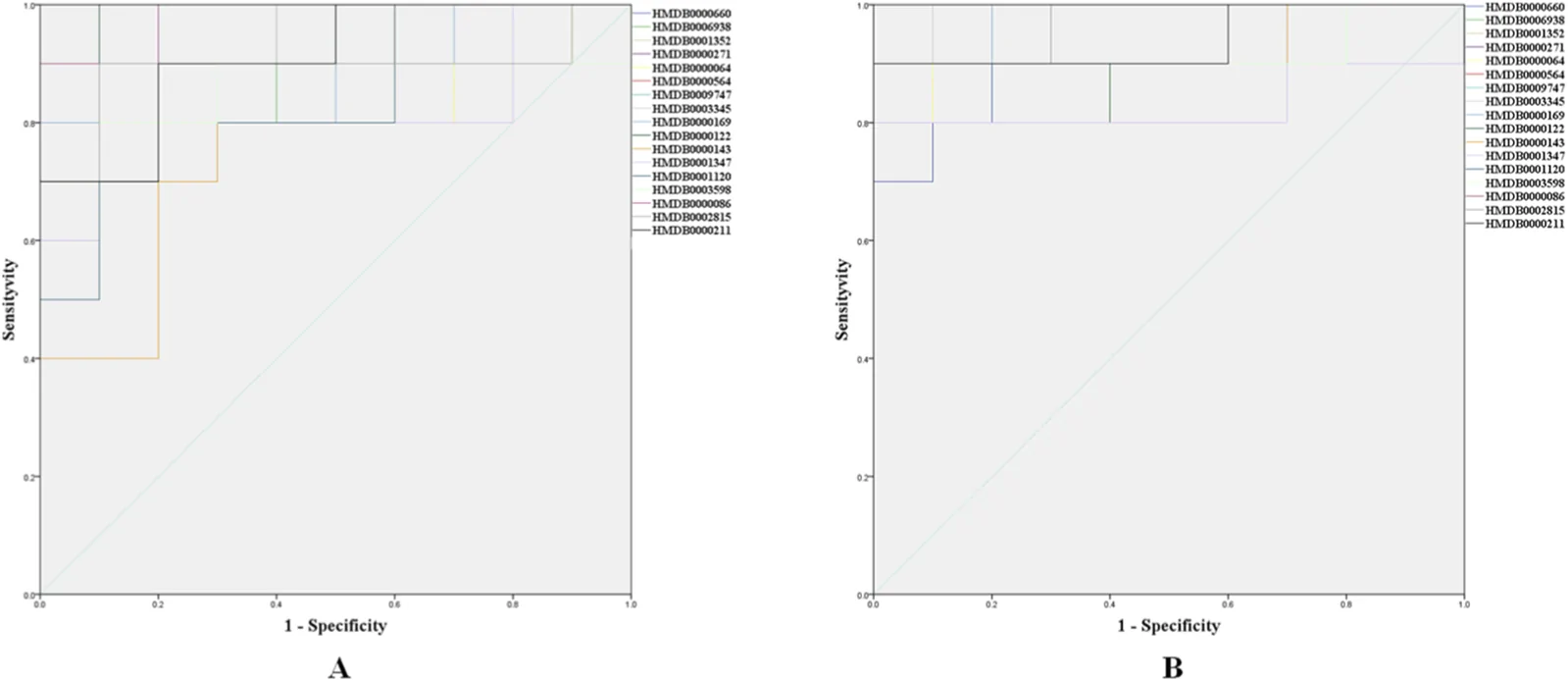
Subject operating curve (ROC), **(A)** Control group vs. Model group, **(B)** FcGH group vs. Model group.

**TABLE 1:** AUC and p-values of biomarkers in the ROC curve.

HMDB	FcGH vs. model		Model vs. control	
	AUC	p	AUC	p
HMDB0000660	0.930	<0.01	0.940	<0.001
HMDB0006938	0.910	<0.01	1.000	<0.001
HMDB0001352	0.830	<0.05	1.000	<0.001
HMDB0000271	0.990	<0.001	1.000	<0.001
HMDB0000064	0.820	<0.05	0.970	<0.001
HMDB0000564	0.960	<0.001	1.000	<0.001
HMDB0009747	0.980	<0.001	0.900	<0.01
HMDB0003345	0.930	<0.01	0.990	<0.001
HMDB0000169	0.880	<0.01	0.980	<0.001
HMDB0000122	0.990	<0.001	0.860	<0.01
HMDB0000143	0.820	<0.05	0.930	<0.01
HMDB0001347	0.820	<0.05	0.830	<0.05
HMDB0001120	0.840	<0.05	1.000	<0.001
HMDB0003598	0.860	<0.01	0.920	<0.01
HMDB0000086	0.980	<0.001	1.000	<0.001
HMDB0002815	0.890	<0.01	0.970	<0.001
HMDB0000211	0.910	<0.01	0.940	<0.001

#### 2.2.3 Biomarker screening and pathway enrichment

The 17 identified metabolites are shown in Table 2. The contents of all metabolites in the Model group, Control group, and FcGH group are displayed in Figure 9. A heat map was constructed for a more intuitive observation of the relative content of all metabolites, as shown in Figure 10A, where blue to red indicates increasing biomarker abundance.

**TABLE 2:** Identified biomolecules.

No.	RT(s)	Compound name	Mass (Da)	Δm (ppm)	Adducts	HMDB ID	Pathway	Change trend	
								M/C	FcGH/M
1	22.651	Isopentenyl pyrophosphate	244.99804	2	M-H	HMDB0001347	TBB	↓	↑
2	22.883	Hydroxypyruvic acid	107.04294	1	M-H	HMDB0001352	GDM,GSM	↑	↓
3	35.5525	PC(32:0)	756.55607	3	M + Na	HMDB0000564	GLM	↑	↓
4	35.96	D-glucose	287.13092	1	M + Na	HMDB0000122	GAM,SSM	↑	↓
5	36.0655	Retinyl ester	301.21627	3	M-H	HMDB0003598	REM	↓	↑
6	36.4235	PE (24:1 (15Z)/15:0)	810.59946	3	M + Na	HMDB0009747	GLM	↑	↓
7	85.501	D-galactose	275.09332	1	M + Na	HMDB0000143	GAM	↑	↓
8	92.11	D-Mannose	185.12798	1	M + H	HMDB0000169	GAM	↑	↓
9	94.667	Tartronate semialdehyde	103.00359	1	M-H	HMDB0006938	GDM	↑	↓
10	97.076	alpha-D-Glucose	175.04728	1	M + Na	HMDB0003345	GAM	↑	↓
11	99.21	Dimethylallylpyrophosphate	240.0178	2	M-H	HMDB0001120	TBB	↓	↑
12	158.8465	LysoPC(18:1/0:0)	522.3531	4	M + H	HMDB0002815	GLM	↓	↑
13	253.011	D-fructose	203.0525	1	M + Na	HMDB0000660	SSM,GAM	↑	↓
14	281.132	Creatine	132.07727	4	M + H	HMDB0000064	GSM	↑	↓
15	281.1365	Sarcosine	90.05493	0	M + H	HMDB0000271	GSM	↑	↓
16	310.371	Glycerophosphocholine	280.09168	2	M + Na	HMDB0000086	GLM	↓	↑
17	314.778	Myo-inositol	258.10968	1	M + H	HMDB0000211	GAM,IPM	↓	↑

RT, retention time (s); Mass, detected mass-to-charge ratio (Da); Δm, relative deviation (ppm); ‘C' control group; ‘M' model group; '↑' upregulated metabolite level; '↓' downregulated metabolite level; ‘PE’: phosphatidylethanolamine; ‘PC’: phosphatidylcholine.

**FIGURE 9:**
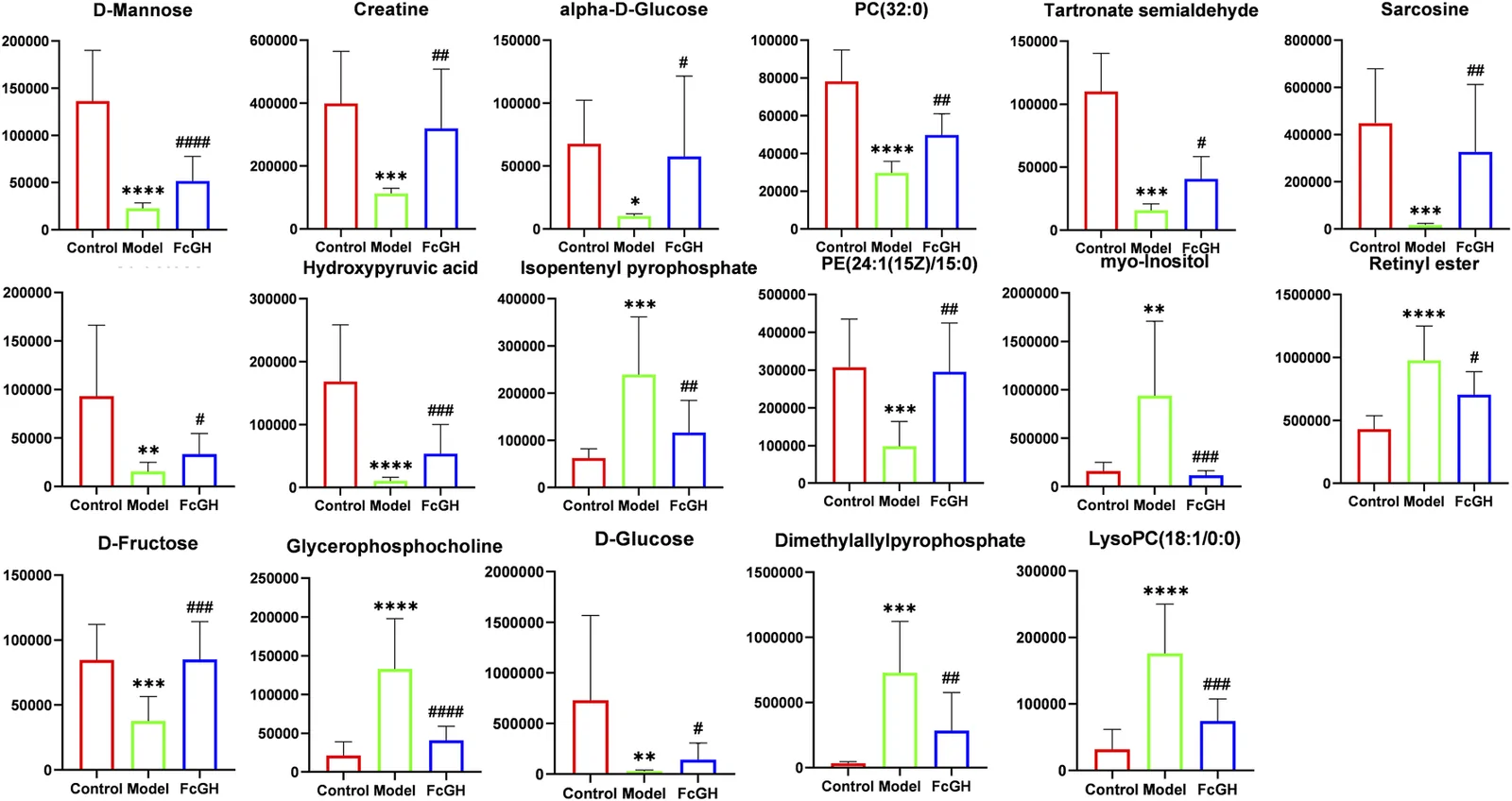
Relative content of different metabolites among the Model group, Control group, and FcGH group, mean ± standard deviation (n = 10). Compared with the Control group: ****p < 0.0001, ***p < 0.001, **p < 0.01, *p < 0.05; compared with the Model group: ####p < 0.0001, ###p < 0.001, ##p < 0.01, #p < 0.05.

**FIGURE 10:**
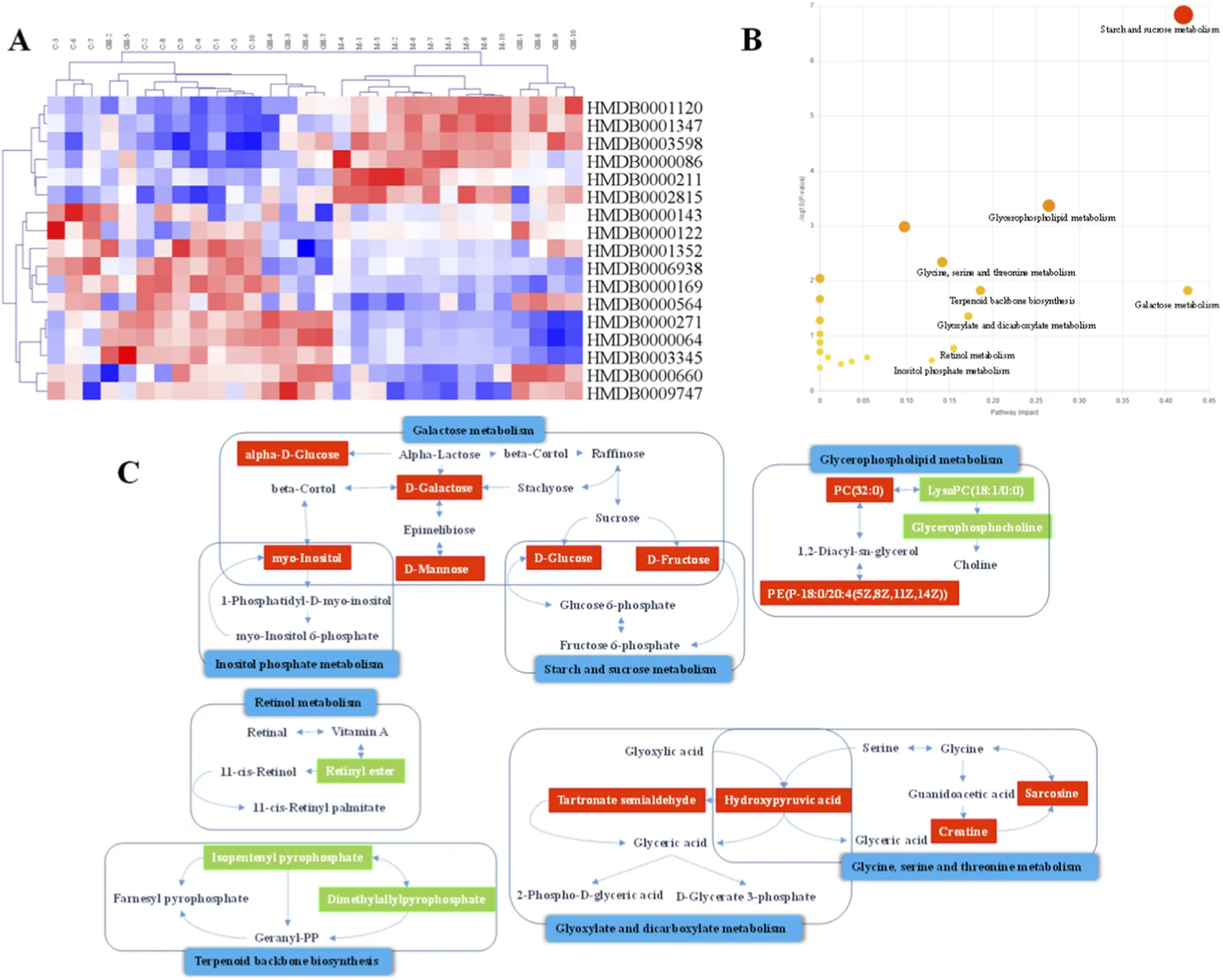
**(A)** Heatmap of biological metabolites, **(B)** Metabolic pathway enrichment analysis, **(C)** Network diagram of biological metabolites and metabolic pathways.

Using the MetaboAnalyst 6.0 platform, pathway enrichment analysis was performed on the 17 identified biological metabolites. The results are presented in Figure 10B and Table 3. Among these, eight pathways with impact values greater than 0.1 were identified, namely,: starch and sucrose metabolism, galactose metabolism, glycerophospholipid metabolism, terpenoid backbone biosynthesis, glyoxylate and dicarboxylate metabolism, retinol metabolism, glycine, serine and threonine metabolism, and inositol phosphate metabolism. Subsequently, a pathway network diagram based on these 17 biological metabolites was constructed (Figure 10C), which more intuitively illustrates the connections between the 17 metabolites and the eight metabolic pathways. In this network, red indicates upregulated metabolite levels, while green indicates downregulated metabolite levels. Collectively, these results suggest that FcGH promotes fracture healing, at least in part, by modulating the metabolic disturbances of fracture-affected biological metabolites within these pathways.

**TABLE 3:** Metabolomics analysis of pathways affecting fracture healing in rats.

No.	Pathway name	Match status	p	−log(p)	Impact
1	Starch and sucrose metabolism	2/18	1.00*10^−1^	0.998786	0.43
2	Galactose metabolism	6/27	1.00*10^−4^	3.999523	0.42
3	Glycerophospholipid metabolism	4/36	2.11*10^−2^	1.6758	0.26
4	Terpenoid backbone biosynthesis	2/18	1.00*10^−1^	0.998786	0.19
5	Glyoxylate and dicarboxylate metabolism	2/32	2.51*10^−1^	0.600655	0.17
6	Retinol metabolism	1/17	4.07*10^−1^	0.390054	0.15
7	Glycine, serine and threonine metabolism	3/33	7.46*10^−2^	1.127477	0.14
8	Inositol phosphate metabolism	1/30	6.04*10^−1^	0.218762	0.13

**TABLE 4:** Instruments and consumables.

Instruments/consumables	Model number/item number	Brand
Mass spectrometer	AB triple TOF 6600 Mass spectrometer	AB SCIEX
Ultra-high performance liquid phase	Agilent 1,290 infinity LC	Agilent
Centrifuge	5430R	Eppendorf
Grinder	JXFSTPRT-CL	Shanghai Jingxin industrial Co., LTD.
Electronic balance	AL104	METTLER TOLEDO
Vortex instrument	QT-1	Shanghai Qite analytical instrument Co., LTD.
Ultrasonic instrument	JP-100	Cleaning equipment Co., LTD.
Chromatographic column	ACQUITY UPLC BEH amide 1.7 μm, 2.1 mm × 100 mm column	Waters
Acetonitrile	1,499,230–935	Merck
Methanol	A456-4	Fisher Chemical
Ammonium acetate	73,594	Sigma
Ammonia water	A470-500	Fisher Chemical

## 3 Discussion

Growth hormone deficiency can lead to skeletal complications and increase the risk of fractures (Criseno et al., 2026). Studies have shown that intervention with growth hormone significantly promotes the recovery of fracture patients (Godoy et al., 2026). There have been no reports on the effect of human growth hormone-Fc fusion protein on fracture healing. In this study, a rat tibial fracture model was established to preliminarily explore the effects of FcGH on fracture healing.

Growth hormone exerts its effects by directly regulating levels of IGF-1 and IGFBP-3 in the body (Cammisa et al., 2026). Studies have indicated that IGF-1 and IGFBP-3 are not only important indicators for predicting fracture risk but also play important roles in the fracture healing process (Michaëlsson et al., 2003; Campos et al., 2022; Wang et al., 2023; Lai et al., 2024). After measuring IGF-1 and IGFBP-3 levels in rats after 4 weeks of medication, significant differences were observed between the model group and the control group, but there were no significant differences compared to the teriparatide group. However, there were significant differences compared to the high- and medium-dose FcGH groups, and these differences were dose-dependent. This suggests that FcGH may promote fracture healing by influencing IGF-1 and IGFBP-3 levels.

In the early stages of fracture healing, mesenchymal stem cells (MSCs) aggregate and gradually form cartilage tissue, with some cells differentiating into osteocytes and gradually undergoing apoptosis (Kronenberg, 2003). During this process, endochondral ossification occurs, and together with the apoptotic osteocytes, gradual calcification forms a hard callus which progressively thickens (Gerstenfeld et al., 2003). Therefore, the size of the callus and cartilage is an important indicator for assessing the degree of fracture healing. Micro-CT results showed that after 2 weeks of medication, all treatment groups had a significantly thicker callus compared with the model group, demonstrating that all drugs promoted fracture healing. After 3 weeks of medication, no visible fracture line was observed in the high-dose group, while clear fracture lines remained in the positive drug group and the medium-dose group, indicating that high-dose FcGH had a more pronounced effect on fracture healing than the positive drug. After 4 weeks of medication, compared with the model group, there were no obvious fracture lines in the teriparatide group, high-dose FcGH group, or medium-dose FcGH group; at the same time, histological sections revealed that by the third week of treatment, cartilage tissues in the high-dose FcGH group had almost disappeared compared to other groups, indicating the best healing effect in that group. After 4 weeks of treatment, the model group and the low-dose FcGH group still showed prominent fracture lines and noticeable cartilage tissue, suggesting incomplete healing, while the teriparatide group and both medium- and high-dose FcGH groups showed disappearance of cartilage tissue and obvious formation of compact bone, indicating good fracture healing. These results indicate that FcGH has a significant effect in promoting fracture healing, most likely by affecting IGF-1 and IGFBP-3 levels.

Metabolomics analysis showed that FcGH influences the fracture healing process by affecting 17 metabolites in eight metabolic pathways.

Galactose metabolism is an important pathway for the synthesis of keratan sulfate and collagen glycosylation in the human body, directly impacting bone development (Conte et al., 2021),and also provides energy for fracture healing by enhancing the oxidation of glucose and fatty acids (Johannsen et al., 2013), inositol phosphate is a precursor of inositol hexaphosphate, whose levels directly influence the synthesis of inositol hexaphosphate (Verbsky et al., 2005), which has been shown to directly affect the body’s absorption of bone minerals (Bahadoran et al., 2025), so inositol phosphate metabolism can directly influence fracture healing. These two metabolic pathways, through inositol, D-glucose, and D-fructose as biomarkers, form a metabolic network with starch and sucrose metabolism, which has the strongest impact, providing an energy source during the regulation of key metabolites in the fracture healing process and thus impacting bone tissue recovery (Zhao et al., 2024).

Clinical studies have shown that glyoxylate and dicarboxylate metabolism directly affects bone density (Aleidi et al., 2021); glycine and serine in the glycine, serine, and threonine metabolic pathway are important risk factors for fractures (Tao et al., 2017; Zhang et al., 2020); these two pathways, connected by hydroxy-pyruvic acid as a key link, form a metabolic network that, by regulating hydroxy-pyruvic acid and other metabolites, can directly affect the course of fracture healing.

Vitamin A is a product of retinol metabolism, with retinyl ester as its main storage form, and its level directly impacts bone quality (Lerner, 2024); both excessive and insufficient levels increase fracture risk, so retinol is also considered a fracture risk factor (Boucher, 2003). Cholesterol, synthesized through the mevalonate (MVA) pathway in the body, is an important product in the biosynthesis of terpenoids, directly participating in the bone remodeling process (Okamura et al., 2010; Hasan et al., 2018).

Glycerophospholipid metabolism is an important lipid metabolic step, and phosphatidylcholine (PC) and phosphatidylethanolamine (PE) are the main phospholipid components of cell membranes, making this pathway crucial for cell membrane formation. Regulating PC and PE levels can directly affect the formation of osteocytes and thereby influence fracture healing in patients (Han et al., 2020; Pihlstrom et al., 2025).

The results show that FcGH can affect eight metabolic pathways by regulating the levels of 17 metabolites, thereby collectively promoting bone fracture healing.

## 4 Materials and methods

### 4.1 Instruments and consumables

The instruments and consumables used in this experiment are listed in Table 4.

### 4.2 Preparation of FcGH injection solution

The FcGH bulk solution produced by Changchun Institute of Biological Products Co., Ltd. is prepared into injectable FcGH by using a 10 mM PB buffer system containing FcGH fusion protein at 12.5 mg/mL, polysorbate 80 at 1–5 mg/mL, disodium EDTA at 0.1–0.3 mg/mL, and sodium chloride at 7–10 mg/mL, with the final pH adjusted to 7.0. In the experiment, the injectable FcGH needs to be used for 0.1% bovine serum albumin saline solution was diluted to 1.8 mg/mL, 0.9 mg/mL, and 0.45 mg/mL respectively.

### 4.3 Effects of FcGH injection on the rat tibia model

Four-week-old, weighing 200 ± 2 g, male, SPF-grade SD rats, purchased from Liaoning Changsheng Biotechnology Co., Ltd., animal license number: SCXK(Liao)2020-0001, with a total of 102 rats. All were housed in an SPF animal laboratory, with an environmental temperature of 25 °C ± 2 °C, humidity of 60% ± 5%, and internal and external pressure differential ≥10 Pa. Throughout the experiment, rats had free access to food. They were adaptively housed for 7 days under the standard specific pathogen-free (SPF) environment. The experimental unit license number is SCXK (Ji)2022-0003, laboratory of Changchun Institute of Biological Products Co., Ltd. All animals had free access to food and water. The animal experiment was approved by the Animal Ethics Committee of Changchun Institute of Biological Products Co., Ltd., and conducted under the guidance of the committee; the license number for animal use is: CCIBP202409-01.

In the first phase of the experiment, 72 rats were randomly divided using a random number table into: Model group, Control group, Teriparatide Injection intervention group (TI) (manufacturer: Xinyi Tai (Suzhou) Pharmaceutical) (20 μg/mL), low-dose FcGH injection intervention group (LOW, 0.45 mg/mL), medium-dose FcGH injection group (MID, 0.9 mg/mL), and high-dose FcGH injection intervention group (HIG, 1.8 mg/mL), with 12 rats in each group. The dose was calculated based on the clinical dose of growth hormone and the dose conversion coefficient (0.63) for rats (Liu et al., 2025). During the dosing phase, the MOD group received only normal saline injections, each FcGH group received weekly injections of 0.2 mL of FcGH injection at the corresponding dose, and the MOD and TI groups received daily injections of the corresponding drugs. All rats were administered via subcutaneous injection. The sample size was determined by power analysis to ensure adequate power to detect significant effects.

In all groups, a rat tibial fracture model was established via surgery to simulate the clinical fracture pathology and assess the efficacy of interventions. The surgery adhered to standardized aseptic procedures to ensure model reliability and animal welfare.

Prior to surgery, rats were anesthetized by intraperitoneal injection of Zoletil®50 (Virbac), and the skin was thoroughly disinfected with povidone-iodine solution to prevent intraoperative infection. Except for the control group, all rats had the midshaft tibia completely exposed, with care to avoid damage to surrounding nerves and vessels to maintain local blood supply. A dental short fine-tooth steel saw was used to transect the midshaft of the tibia horizontally, creating a standard transverse fracture model. Finally, an orthopedic drill was used to bore at the intersection of the medial tibial condyle and the extended line of the tibial tuberosity in the knee joint cavity, and a Kirschner wire was inserted into the tibial marrow cavity until it penetrated the entire tibia, achieving stable fixation of the fracture ends. Postoperatively, the area was disinfected with povidone-iodine solution, and ampicillin sodium injection (Shandong Lukang Pharmaceutical Co., Ltd.) was administered to suppress potential bacterial infections and ensure smooth recovery.

During the intervention treatment phase, the Control and Model groups received only normal saline injections, each FcGH group received weekly injections of 0.2 mL of FcGH injection at the corresponding dose, and the TI group received daily drug intervention at 2.1 μg/kg.

Micro-CT scanning and modeling were performed at weeks 2, 3, and 4 to assess fracture healing. At weeks 2, 3, and 4, four rats from each group were sacrificed, and tibial tissue samples were collected for Safranin O-Fast Green histopathological staining. All rats were sacrificed at week 4, and blood samples were collected for ELISA to detect the levels of IGF-1 and IGFBP-3.

In the second phase of the experiment, 30 rats were similarly randomized into three groups: a model group (Model), a control group (Control), and a high-dose FcGH injection intervention group (HIG), with 10 rats in each group. The modeling procedure and dosing regimen were identical to those used in the first phase. All rats were sacrificed at week 4, and blood samples were collected for blood metabolomics research.

### 4.4 ELISA test

The levels of IGF-1 and IGFBP-3 factors in serum were measured using an ELISA detection kit (Shanghai Sangon Biotech). According to the manufacturer’s instructions, serum samples diluted 100 times were mixed with antibodies and then with markers and chromogenic substrates. The absorbance was measured at a wavelength of 450 nm using a microplate reader, with a dual-wavelength measurement at 630 nm used as the reference.

### 4.5 Micro-CT scanning and modeling

The entire scanning procedure was performed using an inhalation anesthetic machine with isoflurane for induction. The induction concentration was set at 3%–4%, and the oxygen flow rate was controlled at 0.8–1.5 L/min. Continuous ventilation was maintained for 2–3 min until the rats lost their righting reflex. The rats were fixed in a prone position on the scanning bed, with the limbs gently secured using medical tape. The scanning region was positioned 0.5 mm proximal to the distal femoral growth plate and 0.5 mm distal to the proximal tibial growth plate, with a scan length of 1–2 cm. The scanning parameters were set as follows: X-ray tube voltage 80 kVp, tube current 100 μA, scan resolution (voxel size) 18 μm, rotation angle 360°, rotation step 0.5°, exposure time 500 m, and frame averaging of 4. A 0.5 mm aluminum filter was used to optimize beam hardening effects. Each scan took approximately 15–20 min. A thermostatically controlled heating pad was used throughout the procedure to maintain the rats’ rectal temperature at 37.0 °C ± 0.5 °C, and respiratory rate and heart rate were closely monitored to ensure stable vital signs.

### 4.6 Safranin O-Fast green histopathological staining

Bone tissue was decalcified, fixed, embedded, sectioned, dewaxed, and hydrated, then stained using the Safranin O-Fast Green staining kit (Solarbio, G1371). After dehydration, clearing, and mounting, pathological staining sections were prepared. Finally, the slides were observed and imaged under an optical microscope.

### 4.7 Metabolomics research

#### 4.7.1 Metabolite extraction

After the sample is slowly thawed at 4 °C, take an appropriate amount and add it to a pre-cooled methanol/acetonitrile/water solution (2:2:1, v/v/v), vortex mix, ultrasonicate at low temperature for 30 min, let stand at −20 °C for 10 min, centrifuge at 14,000 g and 4 °C for 20 min, take the supernatant and dry it under vacuum. For mass spectrometry analysis, add 100 μL of acetonitrile/water solution (acetonitrile:water = 1:1, v/v) to redissolve the residue, vortex, centrifuge at 14,000 g and 4 °C for 15 min, and take the supernatant for analysis.

#### 4.7.2 Chromatography-mass spectrometry analysis

Chromatographic separation was performed on an Agilent 1,290 Infinity LC UHPLC system using a HILIC column. The column temperature was maintained at 25 °C, the flow rate was 0.5 mL/min, and the injection volume was 2 μL. The mobile phase consisted of A (water containing 25 mM ammonium acetate and 25 mM ammonium hydroxide) and B (acetonitrile). The gradient elution program was as follows: 0–0.5 min, 95% B; 0.5–5 min, B was linearly changed from 95% to 65%; 5–5.5 min, B from 65% to 40%; 5.5–6 min, B from 40% to 95%; and 6–8 min, B was held at 95%. During the entire analysis, the samples were kept at 4 °C in the autosampler. To avoid signal fluctuations, samples were analyzed in a randomized order. Quality control (QC) samples were inserted into the sample sequence to monitor and evaluate the system stability and the reliability of the experimental data.

MS1 and MS2 spectra were acquired using an AB Triple TOF 6600 mass spectrometer. After separation by the Agilent 1,290 Infinity LC UHPLC system, the eluates were analyzed by the Triple TOF 6600 mass spectrometer (AB SCIEX) using electrospray ionization (ESI) in both positive and negative ion modes. The ESI source parameters were set as follows: nebulizer gas (Gas1): 60; auxiliary heating gas (Gas2): 60; curtain gas (CUR): 30 psi; ion source temperature: 600 °C; and ion spray voltage (ISVF): ±5500 V (for positive and negative modes, respectively). The MS1 scan range was m/z 60–1,200 Da, and the MS2 product ion scan range was m/z 25–1,200 Da. The accumulation time for MS1 was 0.20 s/spectrum, and for MS2 it was 0.05 s/spectrum. MS2 spectra were acquired in information-dependent acquisition (IDA) mode with peak intensity threshold-based selection. The declustering potential (DP) was set to ±60 V (for positive and negative modes), and the collision energy was set to 35 ± 15 eV. The IDA settings were as follows: dynamic exclusion of isotope ions within a 4 Da range, and 10 fragment ions were collected per scan.

#### 4.7.3 Data processing

The raw data was converted to. mzXML format using ProteoWizard, and then peak alignment, retention time correction, and peak area extraction were performed using XCMS software. The data extracted by XCMS was first subjected to metabolite structure identification and data preprocessing (missing value filtering: deleting ion peaks with more than 50% missing values; missing value imputation: KNN imputation; data filtering: filtering features with RSD >50%), The Benjamini–Hochberg method was used to control the false discovery rate (FDR), and adjusted p-values <0.05 were considered statistically significant.

The quality of the experimental data was first assessed by comparing overlaid total ion chromatograms (TICs) of the quality control (QC) samples, performing Pearson correlation analysis, and calculating the relative standard deviation (RSD) of ion peak abundances in the QC samples to verify analytical system stability. Subsequent data analyses were conducted after this quality assessment.

Multivariate statistical analysis was performed on all data, and Simca 15.0 software (Umetrics, Malmo, Sweden) was used to visualize the analysis results. Principal Component Analysis (PCA) was used to evaluate the differences between sample groups. The Orthogonal Partial Least Squares Discriminant Analysis (OPLS-DA) model was used to obtain the variable importance values (VIP) that describe the importance of each variable in the group differences, and the validity of the model was validated by permutation testing. Volcano plots were drawn using Origin 2018 software (OriginLab, Northampton, Massachusetts, United States), which visually present the statistical significance and FC differences of each metabolite. Variables with FC of 2 or <0.5, VIP >1, and p < 0.05 were identified as potential biomarkers (Li et al., 2017). The sensitivity of the candidate biomarkers was assessed by receiver operating characteristic (ROC) curve analysis. A biomarker with an area under the curve (AUC) > 0.8 was considered a key contributor to the intergroup differences (Zhou et al., 2022). HMDB (https://hmdb.ca/),KEGG (http://www.kegg.com/) and MetaboAnalyst (https://www.metaboanalyst.ca/) databases were further used to identify biomarkers. By comparing the MS/MS fragment ion peak intensities, the biomarkers were finally confirmed. MetaboAnalyst 6.0 online analysis platform (https://www.MetaboAnalyst6.0.ca/) was used to perform pathway analysis on the identified biomarkers; pathways with an impact value > 0.1 were considered possible metabolic pathways.

### 4.8 Statistical analysis

All data were analyzed using one-way analysis of variance (ANOVA), followed by the Tukey–Kramer *post hoc* test for multiple comparisons between groups. Statistical analyses were performed using GraphPad Prism version 9.5.1 (GraphPad Software, San Diego, United States). Data are presented as the mean ± standard deviation (SD), and p-values <0.05 were considered statistically significant.

## 5 Conclusion

In this study, using a rat tibia model and blood metabolomics technology, the effect of FcGH on promoting fracture healing was evaluated for the first time. The results show that FcGH has a significant effect on promoting fracture healing, which is likely achieved by upregulating the levels of IGF-1 and IGFBP-3 in the blood. Metabolomics studies indicate that FcGH may regulate the levels of 17 blood metabolites in rats, including sarcosine, isopentenyl pyrophosphate, hydroxypyruvic acid, creatine, D-galactose, D-glucose, retinyl ester, PC(32:0), and affects eight metabolic pathways: starch and sucrose metabolism, galactose metabolism, glycerophospholipid metabolism, terpenoid backbone biosynthesis, glyoxylate and dicarboxylate metabolism, retinol metabolism, glycine, serine and threonine metabolism, and inositol phosphate metabolism, thus promoting fracture healing.

This study still has limitations. This experiment was conducted only as a single-center study with a limited sample size. Future studies will be carried out as multicenter experiments to improve sensitivity and reliability. In addition, metabolomics itself has inherent limitations: databases cannot accurately annotate all metabolites, many metabolic pathways have not yet been fully elucidated, and interactions in metabolism may also interfere with detection results and introduce bias.

This study explored the role of FcGH in promoting fracture healing and preliminarily elucidated its possible mechanism of action through metabolomics. These findings provide a basis for subsequent larger-scale multicenter experiments and clinical translational research on FcGH for the treatment of fractures.

## Data Availability

The datasets presented in this study can be found in online repositories. The names of the repository/repositories and accession number(s) can be found in the article/supplementary material.

## References

[B1] Agarwal N. Bell K. R. Ross L. E. Clement N. D. Ralston S. H. Duckworth A. D. (2026). The effects of anti-osteoporotic medication on fracture healing and outcomes: a systematic review and meta-analysis. Lancet Healthy Longev. 7, 100827. 10.1016/j.lanhl.2026.100827 42229491

[B2] Aleidi S. M. Alnehmi E. A. Alshaker M. Masood A. Benabdelkamel H. Al-Ansari M. M. (2021). A distinctive human metabolomics alteration associated with osteopenic and osteoporotic patients. Metabolites 11 (9), 628. 10.3390/metabo11090628 34564444 PMC8466514

[B3] Atalay Y. Gunes N. Guner M. D. Akpolat V. Celik M. S. Guner R. (2015). Pentoxifylline and electromagnetic field improved bone fracture healing in rats. Drug Des. Devel Ther. 9, 5195–5201. 10.2147/DDDT.S89669 26388687 PMC4571933

[B4] Atcı T. Alagöz E. Yaprak Saraç E. Özbay H. Fatih Daşcı M. Acar A. (2021). Effects of different vardenafil doses on bone healing in a rat fracture model. Jt. Dis. Relat. Surg. 32 (2), 313–322. 10.52312/jdrs.2021.72 34145806 PMC8343867

[B5] Bahadoran Z. Mirmiran P. Azizi F. (2025). A prospective association between dietary phytate intake and risk of fracture in middle-aged and older adults: a 12-Year prospective cohort study. Aging Clin. Exp. Res. 38 (1), 12. 10.1007/s40520-025-03267-2 41288864 PMC12748286

[B6] Barvencik F. (2015). Medikamente und Knochenstoffwechsel. Der Unfallchirurg 118 (12), 1017–1024. 10.1007/s00113-015-0109-5 26573290

[B7] Bellido T. (2024). Bisphosphonates for osteoporosis: from bench to clinic. J. Clin. Investigation 134 (6), e179942. 10.1172/jci179942 38488010 PMC10940084

[B8] Bikle D. D. Tahimic C. Chang W. Wang Y. Philippou A. Barton E. R. (2015). Role of IGF-I signaling in muscle bone interactions. Bone 80, 79–88. 10.1016/j.bone.2015.04.036 26453498 PMC4600536

[B9] Boucher B. J. (2003). Serum retinol levels and fracture risk. N. Engl. J. Med. 348 (19), 1927–1928. 10.1056/NEJMoa021171 12736290

[B10] Cammisa I. De Fortuna G. Rulli E. Rigante D. Cipolla C. (2026). Predictors of response to growth hormone therapy in children with idiopathic growth hormone deficiency: a narrative review. Child. (Basel) 13 (4), 545. 10.3390/children13040545 42073123 PMC13114359

[B11] Campos M. S. Volpon J. B. Ximenez J. P. B. Franttini A. P. Dalloul C. E. Sousa-Neto M. D. (2022). Vibration therapy as an effective approach to improve bone healing in diabetic rats. Front. Endocrinol. (Lausanne) 13, 909317. 10.3389/fendo.2022.909317 36060973 PMC9437439

[B12] Chandran M. Akesson K. E. Javaid M. K. Harvey N. Blank R. D. Brandi M. L. (2024). Impact of osteoporosis and osteoporosis medications on fracture healing: a narrative review. Osteoporos. Int. 35 (8), 1337–1358. 10.1007/s00198-024-07059-8 38587674 PMC11282157

[B13] Clark D. Nakamura M. Miclau T. Marcucio R. (2017). Effects of aging on fracture healing. Curr. Osteoporos. Rep. 15 (6), 601–608. 10.1007/s11914-017-0413-9 29143915 PMC6517062

[B14] Conte F. van Buuringen N. Voermans N. C. Lefeber D. J. (2021). Galactose in human metabolism, glycosylation and congenital metabolic diseases: time for a closer look. Biochimica Biophysica Acta (BBA) - General Subj. 1865 (8), 129898. 10.1016/j.bbagen.2021.129898 33878388

[B15] Cooper C. (1997). The crippling consequences of fractures and their impact on quality of life. Am. J. Med. 103 (2a), 12S–17S. ; discussion 17S-19S. 10.1016/s0002-9343(97)90022-x 9302893

[B16] Criseno S. Gayton E. Araghi M. Machtiger-Azoulay N. Toogood A. (2026). Potential impacts of adult growth hormone deficiency: a population-based study in England. BMJ Open 16 (2), e108782. 10.1136/bmjopen-2025-108782 41651512 PMC12887491

[B17] Danowitz M. Grimberg A. (2022). Clinical indications for growth hormone therapy. Adv. Pediatr. 69 (1), 203–217. 10.1016/j.yapd.2022.03.005 35985710 PMC9754707

[B18] Desai B. J. Meyer M. H. Porter S. Kellam J. F. Meyer R. A. Jr (2003). The effect of age on gene expression in adult and juvenile rats following femoral fracture. J. Orthop. Trauma 17 (10), 689–698. 10.1097/00005131-200311000-00005 14600568

[B19] Ding Y. Fan J. Li W. Peng Y. Yang R. Deng L. (2014). The effect of albumin fusion structure on the production and bioactivity of the somatostatin-28 fusion protein in Pichia pastoris. J. Ind. Microbiol. Biotechnol. 41 (6), 997–1006. 10.1007/s10295-014-1440-5 24752560

[B20] Einhorn T. A. Gerstenfeld L. C. (2014). Fracture healing: mechanisms and interventions. Nat. Rev. Rheumatol. 11 (1), 45–54. 10.1038/nrrheum.2014.164 25266456 PMC4464690

[B21] Foulke B. A. Kendal A. R. Murray D. W. Pandit H. (2016). Fracture healing in the elderly: a review. Maturitas 92, 49–55. 10.1016/j.maturitas.2016.07.014 27621238

[B22] Gerstenfeld L. C. Cullinane D. M. Barnes G. L. Graves D. T. Einhorn T. A. (2003). Fracture healing as a post-natal developmental process: molecular, spatial, and temporal aspects of its regulation. J. Cell Biochem. 88 (5), 873–884. 10.1002/jcb.10435 12616527

[B23] Godoy I. R. B. Rodrigues T. C. Yamada A. F. Skaf A. (2026). Atypical “opened-bottle” proximal tibial fractures in young male patients with growth hormone and aromatase inhibitor treatment: case series. Skelet. Radiol. 55 (2), 477–482. 10.1007/s00256-025-05035-9 40928549

[B24] Han Y. Zhang W. Liu J. Song Y. Liu T. Li Z. (2020). Metabolomic and lipidomic profiling of preoperative CSF in elderly hip fracture patients with postoperative delirium. Front. Aging Neurosci. 12, 570210. 10.3389/fnagi.2020.570210 33192460 PMC7642614

[B25] Hasan W. N. W. Chin K. Y. Jolly J. J. Ghafar N. A. Soelaiman I. N. (2018). Identifying potential therapeutics for osteoporosis by exploiting the relationship between mevalonate pathway and bone metabolism. Endocr. Metab. Immune Disord. Drug Targets 18 (5), 450–457. 10.2174/1871530318666180423122409 29683099

[B26] Hixon K. R. Miller A. N. (2022). Animal models of impaired long bone healing and tissue engineering‐ and cell‐based *in vivo* interventions. J. Orthop. Res. 40 (4), 767–778. 10.1002/jor.25277 35072292

[B27] Johannsen D. Kase E. T. Nikolić N. Bakke S. S. Bogen K. K. Aas V. (2013). Remodeling of oxidative energy metabolism by galactose improves glucose handling and metabolic switching in human skeletal muscle cells. PLoS ONE 8 (4), e59972. 10.1371/journal.pone.0059972 23560061 PMC3613401

[B28] Knox A. M. McGuire A. C. Natoli R. M. Kacena M. A. Collier C. D. (2021). Methodology, selection, and integration of fracture healing assessments in mice. J. Orthop. Res. 39 (11), 2295–2309. 10.1002/jor.25172 34436797 PMC8542592

[B29] Kronenberg H. M. (2003). Developmental regulation of the growth plate. Nature 423 (6937), 332–336. 10.1038/nature01657 12748651

[B30] Lai A. Sun J. Dai Z. Guo L. Tao D. Li H. (2024). Unraveling IGFBP3-mediated m6A modification in fracture healing. Pathol. Res. Pract. 255, 155220. 10.1016/j.prp.2024.155220 38432050

[B31] Lerner U. H. (2024). Vitamin A – discovery, metabolism, receptor signaling and effects on bone mass and fracture susceptibility. Front. Endocrinol. 15, 1298851. 10.3389/fendo.2024.1298851 38711977 PMC11070503

[B32] Li Y. Jin Y. Yang S. Zhang W. Zhang J. Zhao W. (2017). Strategy for comparative untargeted metabolomics reveals honey markers of different floral and geographic origins using ultrahigh-performance liquid chromatography-hybrid quadrupole-orbitrap mass spectrometry. J. Chromatogr. A 1499, 78–89. 10.1016/j.chroma.2017.03.071 28390668

[B33] Li G. Chen L. Chen K. (2018). Curcumin promotes femoral fracture healing in a rat model by activation of autophagy. Med. Sci. Monit. 24, 4064–4072. 10.12659/msm.908311 29902161 PMC6032800

[B34] Luo L. Lin J. Ma W. Fan J. (2024). Effectiveness of teriparatide in in improving healing rates and bone-turnover markers of osteoporotic hip fracture: a meta-analysis. J. Pak. Med. Assoc. 74 (4), 741–751. 10.47391/jpma.9245 38751272

[B35] Liu H. Peng B. Zhou B. Zhang Y. Liu Y. Liu Y. (2025). Pharmacodynamics, pharmacokinetics, and toxicology of Fc-growth hormone fusion protein in macaques. Growth Horm. IGF Res. 81, 101648. 10.1016/j.ghir.2025.101648 40120208

[B36] Locatelli V. Bianchi V. E. (2014). Effect of GH/IGF-1 on bone metabolism and osteoporsosis. Int. J. Endocrinol. 2014, 235060. 10.1155/2014/235060 25147565 PMC4132406

[B37] Maghnie M. Ranke M. B. Geffner M. E. Vlachopapadopoulou E. Ibanez L. Carlsson M. (2022). Safety and efficacy of pediatric growth hormone therapy: results from the full KIGS cohort. J. Clin. Endocrinol. Metab. 107 (12), 3287–3301. 10.1210/clinem/dgac517 36102184 PMC9693805

[B38] Menger M. M. Merscher B. Scheuer C. Braun B. J. Herath S. C. Rollmann M. F. (2021). Amlodipine accelerates bone healing in a stable closed femoral fracture model in mice. Eur. Cells Mater. 41, 592–602. 10.22203/eCM.v041a38 34027631

[B39] Metineren H. Dülgeroğlu T. C. Metineren M. H. Aydın E. (2017). Effect of nebivolol on fracture healing: an experimental rat model. Adv. Clin. Exp. Med. 26 (6), 919–923. 10.17219/acem/66291 29068591

[B40] Michaëlsson K. Lithell H. Vessby B. Melhus B. (2003). Serum retinol levels and the risk of fracture. N. Engl. J. Med. 348 (4), 287–294. 10.1056/nejm200305083481917 12540641

[B41] Okamura E. Tomita T. Sawa R. Nishiyama M. Kuzuyama T. (2010). Unprecedented acetoacetyl-coenzyme A synthesizing enzyme of the thiolase superfamily involved in the mevalonate pathway. Proc. Natl. Acad. Sci. U. S. A. 107 (25), 11265–11270. 10.1073/pnas.1000532107 20534558 PMC2895072

[B42] Pihlstrom S. Oghabian A. Maatta K. Legebeke J. Makitie R. E. Campeau P. M. (2025). Transcriptomic and lipidomic profiling provide novel insight into the pathogenesis of monogenic SGMS2-related osteoporosis. JBMR Plus 9 (10), ziaf128. 10.1093/jbmrpl/ziaf128 40978119 PMC12445838

[B43] Selvakumar N. Jose L. Sivanandan H. Chhajed S. S. (2026). Assessment of teriparatide’s effect on post-operative functional outcome and fracture healing. J. Orthop. Case Rep. 16 (5), 341–353. 10.13107/jocr.2026.v16.i05.7300 42131001 PMC13161968

[B44] Sheen J. R. Mabrouk A. Garla V. V. (2026). “Fracture healing overview,” in *Statpearls*. Treasure Island (FL) Ineligible Companies. Disclosure: Ahmed Mabrouk Declares No Relevant Financial Relationships with Ineligible Companies. Disclosure: Vishnu Garla Declares No Relevant Financial Relationships with Ineligible Companies. Treasure Island, FL: StatPearls Publishing Copyright © 2026.

[B45] Tao Y. Chen X. Li W. Cai B. Di L. Shi L. (2017). Global and untargeted metabolomics evidence of the protective effect of different extracts of Dipsacus asper Wall. ex C.B. Clarke on estrogen deficiency after ovariectomia in rats. J. Ethnopharmacol. 199, 20–29. 10.1016/j.jep.2017.01.050 28132861

[B46] Verbsky J. W. Chang S. C. Wilson M. P. Mochizuki Y. Majerus P. W. (2005). The pathway for the production of inositol hexakisphosphate in human cells. J. Biol. Chem. 280 (3), 1911–1920. 10.1074/jbc.M411528200 15531582

[B47] Wang M. Qiu Y. Gao L. Qi F. Bi L. (2023). The impact of IGF-1 on alveolar bone remodeling and BMP-2 expression in orthodontic tooth movement in diabetic rats. Adv. Clin. Exp. Med. 32 (3), 349–356. 10.17219/acem/153956 36421043

[B48] Wang J. Wang H. Wang R. Zhuang Y. Jiang C. Zhao B. (2025). Preclinical investigations of a novel PEGylated long-acting human growth hormone. Drug Des. Dev. Ther. 19, 3497–3507. 10.2147/dddt.S517485 40322033 PMC12049125

[B49] Xian L. Wu X. Pang L. Lou M. Rosen C. J. Qiu T. (2012). Matrix IGF-1 maintains bone mass by activation of mTOR in mesenchymal stem cells. Nat. Med. 18 (7), 1095–1101. 10.1038/nm.2793 22729283 PMC3438316

[B50] Zhang X. Xu H. Li G. H. Y. Long M. T. Cheung C.-L. Vasan R. S. (2020). Metabolomics insights into osteoporosis through Association with bone mineral density. J. Bone Mineral Res. 36 (4), 729–738. 10.1002/jbmr.4240 33434288 PMC8488880

[B51] Zhao G. Liu Y. Zheng Y. An M. Zhang J. Zhang J. (2024). Exploring molecular mechanisms of intra-articular changes in osteonecrosis of femoral head using DIA proteomics and bioinformatics. J. Orthop. Surg. Res. 19 (1), 13. 10.1186/s13018-023-04464-3 38169408 PMC10763026

[B52] Zhou B. Liu J. Wang Y. Wu F. Wang C. Wang C. (2022). Protective effect of ethyl rosmarinate against ulcerative colitis in mice based on untargeted metabolomics. Int. J. Mol. Sci. 23 (3), 1256. 10.3390/ijms23031256 35163182 PMC8836019

